# The Design of a Bioinspired Integrated Total Habitability Instrument for Planetary Exploration: A Review of Potential Sensing Technologies

**DOI:** 10.3390/biomimetics10110742

**Published:** 2025-11-05

**Authors:** Karen Donaldson, Jonah Mack, Yuchen Shang, Ian Underwood, Charles Cockell

**Affiliations:** 1 Institute of Integrated Micro and Nano Systems, School of Engineering, University of Edinburgh, Edinburgh EH9 3FB, UK; j.mack-1@sms.ed.ac.uk (J.M.); ian.underwood@ed.ac.uk (I.U.); 2 UK Centre for Astrobiology, School of Physics and Astronomy, University of Edinburgh, Edinburgh EH9 3FD, UKccockell@exseed.ed.ac.uk (C.C.)

**Keywords:** bioinspired sensing, soft robotics, habitability, astrobiology

## Abstract

One key objective of astrobiology is to investigate and discover if other planetary bodies are habitable. The determination of whether an environment is habitable to known life requires measuring liquid water, CHNOPS elements, other nutrients, and energy supplies. Here we investigate the potential for a single instrument capable of sampling these key indicators: a ‘Total Habitability Instrument’. The proposed instrument would be capable of deployment in diverse environments and provide an integrated set of measurements that together allow for the assessment of the habitability of an environment of interest, such as those of the Moon or Mars. We explore existing and potential technological developments that would enable the construction of such an instrument, with a focus on soft systems, which are inspired by nature in their design, and microfluidics. This paper considers a multidisciplinary approach to the design and sensing requirements of a Total Habitability Instrument that would be capable of gathering and processing samples and be deployable by both robotic and human explorers on all planetary bodies, allowing for the mapping of habitability over large areas of our Solar System and beyond.

## 1. Introduction

Habitability is core to astrobiology. Although exact definitions can vary, habitability is a measure of the potential of an environment to sustain life. Provided that an environment is capable of supporting one or more forms of life, it falls under the definition of a habitable space, and therefore, all biology must exist within this space. One of the key interests of astrobiology is the extent of habitable space in the universe and whether such spaces contain life [1,2,3].

A number of requirements must be met for an environment to be habitable. For known life, these are the presence of water, an energy source, the presence of key elements for macromolecular construction, along with physical and chemical conditions that allow for growth or reproduction.

The mere presence of these factors in one environment does not demonstrate habitability. For example, it is possible for liquid water to exist but for the water activity or chaotropicity to be so low that it is uninhabitable [4]. Therefore, with respect to liquid water, the presence of liquid water does not demonstrate the potential for habitability. For this reason, all these parameters must be measured in an environment to define a place as being habitable. This leads directly to the need to develop instrumentation to measure these different physical and chemical conditions within an environment.

The key to assessing habitability is the ability to identify, analyse, and quantify these fundamental indicators, which depends on reliable remote sensing technology. Often, the requirement for in situ measurements is high levels of automation and robust transmission of results. Coupled with the high cost of accessing environments of interest, this poses a set of unique and demanding requirements on instrument design.

Organisms have evolved to adapt and operate in a variety of different extreme and dynamic environments. When considering the use of robotic systems, system compliance can be crucial for their integration into these environments. Hard robots are regularly ‘non-collaborative’ and can require predictable situations and accuracy. However, due to the design and control of soft robotics, they are inherently compliant. Such systems could provide a solution to the required instrument design.

In addressing this challenge, astrobiologists are aided by novel sensing technology provided by advances in related fields such as microfluidics, biomedical devices, soft systems, and environmental sciences. Development in micro- and nano-fabrication, new sensing modalities, along with the discovery of new materials, has led to exponential improvements in sensing capability [5]. Currently, there are many sensors to detect an equally diverse range of chemical analytes [5]. However, sensor research can often be conducted across disparate disciplines. Considering the importance of sensing to astrobiological research, there is a need to collect and examine the state of the art in sensing technology with a view to habitability.

It is critical to understand the requirements, limitations, and design of a single instrument that would be capable of gathering and processing samples in dynamic environments. A THI system can be deployed by robotic and human explorers and allows for the mapping of habitability markers over large areas.

This review investigates the technology and science behind soft robotics and bioinspired systems to propose solutions to mission mass limitations. Understanding how to reduce the size and weight of these instrumentation system sensors is required. From the diversity of measurements required within extreme environments, and from the advances made in soft robotic manufacturing, locomotion, and embedded systems, the integration of a THI remotely deployed by a soft robotic system would enable adaptable and diverse sensing opportunities.

We review the state of the art in detecting the components of habitability to explore the prospects of a ’Total Habitability Instrument’ (THI), which would allow for the detection and mapping of habitability in different planetary environments.

## 2. Habitability in Aqueous Environments

The discussion in this review is limited to Life “As We Know It” (LAKI), which covers several aspects from the origin of life: the evolution of prokaryotes to eukaryotes, including bacteria, algae, and fungi [6]. Hypotheses about the existence of unfamiliar life with exotic biochemistries not represented in known life are interesting; however, with no empirical evidence for such biochemistries, what constitutes a habitable space can only be scientifically defined by a known point of reference. For all known life, the presence of liquid water is necessary as a solvent for the electrochemical reactions that sustain life and is taken as the baseline assumption for deriving sensor requirements.

### 2.1. Water Bulk Properties

The amount of thermodynamically available water, measured as water activity (*a_w_*), is a limiting factor for all life. While most microbes and fungi are not active at *a_w_* < 0.91 and *a_w_* < 0.7, respectively, organisms have been observed at much lower water activities [7]. The theoretical limit on water activity for all domains of life has been proposed to be around 0.6; however, cell division has since been observed at *a_w_* = 0.585.

Water activity illustrates that the line between habitable and uninhabitable is very fine; therefore, sensors must have suitably high resolution and accuracy to determine these critical interface conditions.

### 2.2. Ionic Environment

In addition to water activity, ionic strength, which is a measure of the charge density in a solution, can limit the water activity and, therefore, the habitability of an environment. The presence of ions is necessary for life on Earth. Major cations Mg^2+^, Na^+^, Ca^2+^, and K^+^ have varying but essential functions in microbial life. For example, potassium is an abundant monovalent cation that plays a major role in the cell, such as the regulation of osmotic pressure and pH control [8].

Chaotropic (disordering) salts such as MgCl_2_ have a limiting effect on biology by working to denature biological macromolecules [4]. High concentrations of chaotropic species have been found to be a limiting factor for life both in terrestrial subsurface environments [9] and in simulated Martian brines [10]. Research has been conducted on brine-saturated Martian analogue materials [11,12]. Thus, measurements of ions can not only confirm the presence of bioessential ions but also provide an assessment of the chemical limits to life.

One particular ion of importance, which can define the diversity of life in an environment, is the concentration of H^+^ ions (H_3_O^+^). H^+^ ion measurements are defined as the ‘potential of hydrogen’ (pH) scale. Both extremes of the pH scale can present challenges to biology. Although no environment on Earth is known where extremes of pH alone are a barrier to life, pH can affect the diversity of potential life and, by influencing the chemical state of other ions, indirectly establish boundaries for habitability. A low pH denatures proteins and is hostile to a wide range of organisms. However, microorganisms known as acidophiles have been found to reproduce in solutions with pH values below neutral [13]. These include fungi, eukaryotes, and archaea [14]. At the other extreme, alkali conditions host alkaliphiles, which are found in naturally occurring soda lakes, with a pH of 13 [15].

Therefore, it is not only the presence of certain chemicals important to life that determines habitability but also the concentration. A sensor for habitability must be able to detect and quantify a wide range of necessary ions. Extreme pH values, as well as environments with high ionic strength, can detrimentally affect the performance of many sensors. These effects must be taken into account in sensor design.

### 2.3. CHNOPS

The elements of “CHNOPS”: carbon (C), hydrogen (H), nitrogen (N), oxygen (O), phosphorus (P), and sulphur (S) are common to all known life [3,5] and the requirements for habitability. As both hydrogen and oxygen are the constituent elements of water, molecular oxygen acts as an electron acceptor for many biological redox reactions [16].

CHNOPS elements can be present as compounds (e.g., phosphorus in calcium phosphate); however, where a liquid exists, they may be in dissolved ionic form. Microbes can access these elements from solid form, by, for example, acidification of their local microenvironment [17]. In this review, we focus on habitability detection in aqueous environments and, therefore, the detection of these elements in the ionic state. When developing the design and sensor selection for a THI, the exact expected CHNOPS elements should be considered to inform the range of sensors integrated.

Due to their ubiquity and importance to known life, the CHNOPS elements have been, alongside liquid water, the subject of the greatest interest in space missions within the Solar System [5].

### 2.4. Energy

A suitable energy source is a necessary requirement for habitability [18]. Microbial life in Earth’s deep biosphere provides evidence of metabolism in relatively energy-starved conditions [19]. Redox reactions are universal in the supply of useful energy within the organism to maintain its own chemical reactions [20]. There exists a wide diversity of redox processes available to known life [21]. Requirement: the system must be able to identify and quantify the most likely of these redox elements and compounds present in an environment.

### 2.5. Physical Extremes to Life: Temperature

Life must operate within other physical and chemical boundaries suitable for biological processes. One of these conditions is the boundaries set by the temperature limitations. The availability of liquid water places a firm limit on temperature under certain pressures and ionic concentrations. The physical phase space for the existence of liquid brine extends well beyond that of pure water. At the lower end, the possible existence of liquid perchlorate brines on Mars at temperatures down to −65 °C [22], while the phase diagram of water shows that liquid water can exist at temperatures of 300 °C at 10 MPa. The limits of habitable temperature are likely to be constrained to a much tighter range. To date, the highest temperature at which growth has been observed is 122 °C [23], while the lowest temperatures for growth and metabolic activity are −15 °C and −25 °C, respectively [24].

Sensing technologies for temperature are well established and have been thoroughly reviewed [25]. Research has made considerable advances on temperature sensors with specific applications [26] and materials [27,28]; therefore, sensors that are accurate, inexpensive, low-power, and highly miniaturised are widely commercially available.

### 2.6. Polyextremes

Environments rarely exhibit only a single extreme. Environments with high ionic strength often exist at high- or low-temperature extremes, for example, Siberian cryopegs [29] or hyperacidic, hypersaline, and high-temperature regions of Dallol hot springs [30,31]. These multiple chemical and physical extremes, known as polyextremes, often influence biology in complex ways. For example, while chaotropic solutes have been found to be detrimental to life, in combination with low-temperature extremes, they have been found to increase the window for habitability [32]. Other studies of polyextremophiles have been conducted in the Red Sea brines [33], deep-sea hydrothermal vents [34], and the Salar de Huasco basin in Chilé [35]. These represent distinct examples in the limits of habitability. A review by Harrison et al. notes the influence of the polyextremes of temperature, pH, salinity, and pressure on the growth of prokaryotic strains [36]. Pressure can act in synergy with other extremes to limit life, and it is useful to measure this parameter. High pressures are known to influence the biochemistry and biophysics of life [37], and low pressure can also act as a limit to life [38]. Thus, pressure sensing allows us to assess the combined set of extremes to which life would be exposed.

It is important that habitability detection takes into account these multiple extreme conditions. In order to map any potential habitable environment, there should be a capability to determine where it lies in the phase space of habitability. The habitability phase space refers to the set of combined conditions that would allow for certain organisms to be metabolically active or to reproduce. Once data from the detectors is gathered, the combined data can be examined against the requirements and limits of known organisms. As combinations of extremes can act synergistically or antagonistically [36], often a single habitability index cannot be calculated but rather the data must be assessed against existing biological data.

## 3. Sensing in Aqueous Environments

A sensing system that detects all or most of the important parameters for habitability can be termed a Total Habitability Instrument (THI). Its function would be to determine whether an environment is habitable, and if so, where in the habitability phase space it lies.

A THI would not only be a fully integrated array of different sensing modalities. Inspiration can be drawn from adjacent fields such as environmental science, where there is research in the development of micro-total analysis systems (*µ*TAS) [39,40]. Much of the current research on *µ*TAS in astrobiology is focussed on organics and the search for biomarkers [41,42,43,44].

The following section examines technologies for the measurement of each habitability condition, comparing novel methods to established instruments to provide an overview of the key features required to sense the parameters, while highlighting examples of sensors with potential use in a THI.

### 3.1. Water Bulk Properties

Prior to the deployment of a THI, water may well have been detected by remote sensing technologies [45,46]. Earth-based infrared spectroscopy [47], images from the Voyager spacecraft [48], and Doppler and high-resolution images from Galileo [49,50] provide some of the evidence for subsurface oceans on Europa. Data suggesting the existence of subglacial water bodies on Enceladus is provided by Cassini’s flyby and analysis of large plumes of liquid water with its on-board Ion and Neutral Mass Spectrometer (INMS) [51,52]. These technologies inform about the bulk presence of water; the THI should be capable of investigating the habitability of these diverse aqueous environments.

### 3.2. Water Activity

Water activity in liquid samples is conventionally measured by enclosing the liquid within a fixed volume and measuring the relative humidity in the gas phase once vapour–liquid equilibrium is reached. Sensors measure relative humidity using a wide range of techniques, transduction methods, and materials, each with trade-offs in cost, operating range, and sensitivity [53,54,55].

Water activity can also be measured optically, using materials with properties sensitive to changes in equilibrium relative humidity (ERH). Notably, optical grating fibres, coated with a hygroscopic material, such as polymers [56,57], gelatine [58], or graphene-oxides [59], have been explored as effective sensors. The advantages of these sensors over traditional capacitive/resistive humidity sensors provide improved tolerance to harsh conditions and insusceptibility to electromagnetic interference [60].

In selecting an appropriate technology for measuring habitability, several factors must be considered. The working range of an ideal sensor would allow accurate water activity measurements of 0.5 > *a_w_* > 1, which would account for the full habitable space, as well as extending comfortably beyond the threshold. However, as *a_w_* approaches 1, it is no longer a barrier to habitability for most life. Thus, accuracy throughout the ideal range is less important than that at the interface, which occurs around 0.9 and 0.7 for microbes and fungi, respectively [7].

As discussed previously, the line between habitable and uninhabitable spaces is very fine. A difference of ±0.1 *a_w_* is biologically significant, especially at the interface of the water activity limit, and would considerably alter the ability of an environment to sustain life. Any sensor must therefore have the appropriate resolution and precision to characterise an environment firmly in the habitability phase space.

Water activity measurements using conventional methods are well established and bring with them the advantages of robust and reliable measurement. However, certain features pose challenges for integration with other sensing modes and miniaturisation. The need for measurement in the gas phase within an enclosed volume necessitates a space separate from other sensors, which may require direct contact or submersion within the liquid itself. Whilst technologies exist that can measure within the liquid phase, they have commonly been developed for dual use in another sector. An example of this is a microelectromechanical system (MEMS)-based tensiometer. This method uses thin-film fabricated strain gauges to measure changes in tension on the surface of a micro-cavity of liquid water, which in turn is determined by the water activity via the water potential [61]. Measurements in the liquid phase are achieved by packaging the sensor with a hydrophobic material, which maintains a small air gap between the sensor and the liquid in the environment. The use of this type of hydrophobic material could potentially reduce the measurement volume of other sensing methods. Despite successful efforts in lowering the threshold [62], tensiometry remains an unsuitable method for studying habitability due to its restricted range below *a_w_* = 0.9. Such a sensor would be able to report water activity measurements at 0.1 increments, and ideally at a higher resolution than this (down to 0.01 unit increments).

### 3.3. Ionic Strength

The ionic strength is the total ionic content of a solution. This is commonly measured by the electrical conductivity, which is positively related to the ionic strength of a solution. If a voltage is applied to electrodes in a solution, the conductivity can be measured by the resulting current flow. The geometry and separation between the electrodes determine the cell constant, which is related to the measured conductance:

*σ* = *kC*

where *C* is the conductance [S], *k* is the cell constant [m^−1^], and *σ* is the specific conductivity [S m^−1^].

The specific conductivity is often used as it accounts for the cell constant, which varies depending on the instrument used.

Conductivity probes have found various uses within environmental monitoring [63] and are widely used by oceanographers as a measure of salinity. Thin-film microfabrication techniques are used to make planar conductivity sensors [64], which allow them to be easily integrated with other sensing modalities. Microfabricated conductivity sensors have been integrated with temperature and pressure sensors for depth sensing [65,66], as well as pH for multi-parameter sensing in water systems [67]. Novel electrode design and geometry have been reported to produce sensors with higher precision and accuracy for a wider, higher conductivity range [68].

In studying habitability, carbon conductivity probes were used in the Phoenix Lander’s on-board wet chemistry lab [69]. A conductivity temperature probe has also been designed for use and tested in the hot springs of Yellowstone National Park [70]. Conductivity electrodes are a mature technology and are already in established use for sensing salinity in oceanography and environmental studies. Planar sensors are easily integrated with many of the other sensors discussed here, as well as microfluidic sample delivery/processing capabilities. This makes them a highly attractive candidate for use in a habitability sensor.

### 3.4. Ionic Environment

As well as defining the limits to life through water activity and ionic strength, ions may come in the form of CHNOPS elements, micro-nutrients such as transition metals (Fe, Cu, etc.), and the special case of H^+^ ions that define the pH. In addition, the major cations Na^+^, K^+^, Mg^2+^, and Ca^2+^, along with anion Cl^−^, have been found to be essential for many biological processes [71]. Trace ions such as Zn^2+^ and Cu^2+^ also play important, occasionally interchangeable roles in biology.

Therefore, a key consideration in the design of a THI is the characterisation of the ionic environment of a solution. Two broad categories of electrochemical sensing can be employed to achieve this: potentiometric and redox sensing. In the case of potentiometric, ions of inert species are analysed by measuring an ionic activity-dependent boundary potential relative to a reference electrode in solution. In this case, no electrochemical reaction occurs, and the solution remains largely unchanged. In the case of redox sensing, techniques such as voltammetry or amperometry can be used, where an electrochemical reaction is induced, usually through electrodes in contact with the solution. These methods are commonly used for sensing species with a lower redox potential, such as Fe and Cu.

Ionic species that encompass the major bioessential ions, such as the group I and II metals, are largely sensed using potentiometric methods (Table 1), and species that make up the trace essential ions and act as redox pairs for biological redox reactions can be sensed using voltammetry or amperometry.

#### 3.4.1. Potentiometric Sensors

The two most common forms of potentiometric sensors are ion-selective electrodes (ISEs) and a class of Field-Effect Transistors (FETs) known as ion-selective FETs (ISFETs). Though their underlying transduction principle is different, both make use of an ion-selective membrane (ISM) to sense ionic activity. Masullo et al. designed a high precision single-molecule localization scheme (ISM-FLUX) using an ISM with a single-photon avalanche diode (SPAD) array detector to boost the performance of state-of-the-art single molecule tracking experiments in living cells [79]. Glass-membrane electrodes for sensing pH are also potentiometric sensors, with membranes made of glass which respond to pH in solution which function in a similar method as other ISEs, Table 2 shows a comparison of sensing methods.

An ISE consists of an ion-selective membrane (ISM) connected to an electrode, commonly through a liquid junction or a solid contact. When measured against a reference electrode in the same solution at temperature T, the resulting potential difference E is described by the Nernst equation.

$$E=E_{0}+\frac{RT}{zF}In\;a_{i}$$

where *E*_0_ is a potential constant, *z* is the atomic charge of the ion, and *R* and *F* are the gas and Faraday constants, respectively.

The Nernst equation results in a well-defined relationship between the measured potential and the ion activity, which slopes 59 mV/z per decade at a constant temperature. Sensitivity for specific ions is achieved by selecting the ionophore to be doped in the ISM. There are a wide range of ionophores for sensing various ions of interest [80,81,82].

A traditional ISE contains a liquid internal filling solution, which stabilises the interfacial potential at the electrode and membrane boundaries by ion-electron exchanges. This stabilising action is necessary for reliable sensing; however, the liquid filling solution presents several issues. Evaporation or leaching from the sensor increases the required level of maintenance. Additionally, liquid cavities can be challenging to miniaturise; thus, the filling solution becomes a barrier to miniaturisation. There has since been a significant drive to replace it with a solid material with the same stabilising effect, with a common term for these electrodes being solid contact ion-selective electrodes [80].

The earliest solid contact ISEs were simply Ag/AgCl wires coated with an ion-selective membrane [83]. Termed “coated wire electrodes”, these sensors suffered significant drift in short time periods due to the lack of an internal reference and the eventual formation of a water layer. Efforts have been made to stabilise the interfacial boundary potential through the use of various conducting polymer membranes as a replacement for the well-defined ion–electron exchanges enabled by the filling solution [84,85,86].

An alternative approach has been adopted that uses materials with a large double-layer capacitance and high hydrophobicity as ion-to-electron transducers. The large capacitance allows for the fast transfer of electrons, while the hydrophobicity inhibits the formation of the aforementioned water layer. These include platinum nanoparticles [87,88], graphene [89], and various carbon nanomaterials [90,91]. Carbon materials have also been used in ISEs for measurements at high pressures [74].

The dynamic range of ISE is limited by the saturation of ionophore sites in the upper limit and by the movement of ions from the membrane to the solution in the lower limit. For example, the lower detection limit of ISEs has been reported to be down to 10^−10^ M in a Ca^2+^ electrode [92]. As such, ISEs are prevalent in biomedical sensing [93], where their low limit of detection and scope of miniaturisation are critical. They are also attractive for in situ environmental monitoring of water bodies due to their small size and low power consumption [94]. In studying habitability, the most notable example of the use of ISEs to date was on board the Phoenix Lander’s wet chemistry lab, which featured a suite of electrodes [69], measuring a wide range of ions present in the Martian regolith. This is an early example of integrated electrochemical sensors for measuring ionic environments in extraterrestrial settings and lays the groundwork as further research is conducted on developing the next generation of ISE arrays [95].

The development and improvement of miniaturised ISEs is a well-established and highly active field of study, and there exist a wide variety of innovations and designs [80,96,97]. ISEs would therefore be a viable technology for the ionic sensing requirements of a Total Habitability Instrument.

An alternative sensing method to the ISE is the ISFET, which uses the properties of the conventional FET as the underlying transduction method [98]. These sensors are modified metal–oxide–semiconductor FETs (MOSFETs) commonly used in electronics, where an ion-selective membrane potential measured against a reference electrode provides the gate voltage, in turn producing a current proportional to the activity of the species. Utilising complementary metal–oxide–semiconductor (CMOS) processes developed for the semiconductor industry, the underlying FETs of the ISFETs can be mass-manufactured with reduced associated costs. pH sensing may be achieved fully through the CMOS process [99], though post-processing is necessary for sensitivity to other ions. As such, pH sensing has become one of the most common applications of this technology.

ISFETs have the advantage of being chemical sensors already integrated with electronic elements, making them particularly useful in environments with heavy background noise, for example, in medical biosensors [100]. ISFETs are much smaller than traditional ISEs with liquid inner fillings and are easily integrated into 2D planar arrays of sensors with individual addressability, allowing applications such as ion imaging with high spatial resolution [101,102]. This capability makes ISFETs a suitable candidate for multi-ion sensor chips in a THI, with a novel sensor design [73].

However, certain challenges must be addressed for the robust deployment of ISFET sensors. ISFET responses have been reported to show variations with temperature, as temperature affects both the mobility of charge carriers and the threshold voltage in the device [103]. Additionally, ISFETs exhibit significant current drift over time, which is not easily compensated for as it is a function of the size and shape of the sensor [104], as well as the pH, ionic environment, and temperature of the sensing solution [105]. Addressing this issue remains an active field of study, with proposed solutions ranging from external temperature compensation circuits [106] to machine learning [107]. The challenges and applications of ISFETS are reviewed by Bergveld et al. [108] and, more recently, by Moser et al. [109].

#### 3.4.2. pH Sensing

As an important factor across a wide range of fields and disciplines, pH sensors were some of the earliest electrochemical sensors to be developed. First reported in the literature in 1909 [110], the glass pH electrode remains one of the most widely used electrochemical sensors to date. Interest in improved spatial resolution in sampling has driven the development of microelectrodes [111]; however, these designs are brittle and have a limited set of applications.

This issue, along with other challenges faced by traditional glass-membrane electrodes, has largely been addressed with advances in materials sciences and microfabrication techniques. Notably, the ISFET, introduced in Section 3.4.1, was an early innovation in this field. The use of ISFET-based pH sensors has been particularly successful in systems for monitoring seawater [112], with comparable accuracy, improved response time, and lower power consumption to the glass-membrane benchmark [113]. Currently, there is a wide selection of these sensors available commercially, with some showing promising signs for stability and reliability, even when deployed at considerable depths and durations [114]. However, some studies have shown that some commercial ISFETs do not provide adequate capabilities when benchmarked against spectrophotometric methods, citing issues with calibration as a main reason for the shortcomings [115].

These sensors are optimised for sensing in seawater; however, habitable environments can be found across the full pH range. While the most common ISFET applications do not require sensing in the more extreme pH ranges, common metal–oxide coatings can be designed for good pH responses from pH 1 to 13 [116], and changes to the sensing material can produce sensors with an even wider range [117].

In addition to ISFET-based sensors, ISEs based on pH-sensitive metal–oxide films constitute a distinct design class of potentiometric sensors [78]. The most common of these is iridium oxide (IrOx), notable for its high stability and speed of response. IrOx-based pH microelectrodes were initially fabricated in the 1970s; however, since then, technology has allowed for the fabrication of planar electrodes [118,119], which are more readily integrated with other ion sensors [67,120].

#### 3.4.3. CHNOPS Elements

Mass spectrometers (MSs) have been the instrument of choice for the analysis of mineral and atmospheric composition, and therefore, the determination of CHNOPS content. Mass spectrometers have been a staple of scientific space exploration, with instruments being flown on the Apollo [121], Viking [122], Curiosity [123], and Cassini–Huygens [124,125] missions. MSs will continue to be deployed, for example, in the ExoMars [126], Jupiter Icy Moons Explorer (JUICE) [127], and Europa Clipper [128] missions. Since the late 1990s, research has been conducted to reduce the size and mass of MS systems for applications in extraterrestrial exploration and air quality monitoring [129].

Next-generation instruments, such as the Orbitrap [130], have been tested for use in relevant astrobiology analogues and are candidates for flights on future missions [131]. There are, however, limitations to this technology. Miniaturisation is inhibited by their complex operating principle, requiring several stages of sample preparation and manipulation. Kerman et al. [132] designed and tested a miniaturised spectrometer for Raman spectroscopy for a new innovation in sensing. By the same principle, MS remains expensive, particularly for extraterrestrial applications, as each instrument is often custom-built for each specific mission [133]. In addition, MS is a destructive technique and requires the extraction and vaporisation of samples [134].

To address some of these issues, optical spectroscopy techniques have been pioneered to complement MS capabilities. The Mars Chemistry and Complex (ChemCam) spectrometer was the first of its kind to be used in an extraterrestrial setting [135]. The instrument used laser-induced breakdown (LIBS) of minerals to determine their composition to locate areas of interest to extract and analyse samples using the rover’s instruments. LIBS provides a novel way of analysing the chemical composition of targeted soils or rocks; the quality and repeatability of the results are affected by the terrain and soil conditions as a result of physical matrix effects due to varying properties like thermal conductivity. These physical and chemical matrix effects cause difficulties with quantitative LIBS analysis. Moreover, the diverse areas in which LIBS is used can require varying conditions of ablation techniques. Therefore, it is useful to research the effect of different soil characteristics on the ablation process. Research by Donaldson et al. presented a finite element modelling simulation-based investigation on soil quality analysis using LIBS to gain insight into the soil breakdown process, laser coupling, and sample temperature [136]. Yan et al. conducted research to assess the effects of sample pretreatment on the measurement of nitrogen in soil using a mobile LIBS system with an Nd:YAG laser [137].

Raman spectroscopy has long been a candidate for mission instrumentation [138,139]. Examples of Raman instruments have been deployed for in situ measurements of seabeds and sediments [140]. These interfaces are interesting regions for habitability, as mineral water interactions provide much of the chemical requirements for habitability. Beyond this, Raman spectrometers have also been designed for life detection [141].

ESA’s ExoMars rover features a Raman spectrometer for the analysis of powdered samples extracted by the rover’s drills [142]. Following the initial analysis, selected samples analysed by the instrument will continue to be analysed through the Mars Organic Molecule Analyser (MOMA) mass spectrometer. It is designed for in situ analysis of organics from the Martian regolith to identify a wide range of organic molecules including amines, amino acids, aldehydes, ketones, organic acids, thiols, and polycyclic aromatic hydrocarbons (PAHs) with sub-part-per-billion sensitivity [143].

NASA’s Mars 2020 mission’s Perseverance rover features two Raman spectrometers, coupled with LIBS in the SHERLOC [144] and SuperCam [145] instruments for the analysis of minerals as candidates for sample return.

In comparison to mass spectroscopy, remote Raman instruments are able to survey extensively and operate at a long range. The combination with optical imaging provides spatial reference to areas under analysis, and a greater capacity for miniaturisation frees the payload capacity and lowers the launch cost. However, these instruments remain limited in size reduction by the need for optical components. Raman spectroscopy is also less efficient in optically transparent samples; therefore, an analysis of clear liquid or gaseous samples requires significant design modification [146]. Furthermore, although Raman spectroscopy can be used to identify certain minerals, it is not always possible to use this method to identify the full range of ions/CHNOPS elements or quantify their abundance.

In aqueous environments and samples, other methods have the potential to occupy sensing niches that are more challenging for the mentioned instruments to access. Research has been conducted on the possibility of detecting and analysing organic molecules using capillary electrophoresis (CE). The proposed Enceladus Organics Analyser (EOA) instrument couples CE with laser-induced fluorescence (LIF) for ultrasensitive (300 zeptomoles in 1 nL samples) detection of a wide range of organic molecules [44].

CE has several advantages for the analysis of organic compounds. Coupled with LIF, CE methods have the lowest limit of detection among the methods discussed in this section. The extraction of analytes from solids is achieved non-destructively using liquid extraction. Of particular interest to this paper, samples are taken in the liquid phase—allowing direct extraction from an aqueous environment. Furthermore, CE has a high potential for miniaturisation, demonstrated in the manufacture of a low-cost (ca. USD 500), miniaturised LIF device [147].

In determining inorganic CHNOPS composition, research in environmental sciences offers analogous technologies that can be applied to the field of astrobiological analysis. Current research trends towards in situ, autonomous, and miniaturised sensors [148,149]. In this application, CE is again a focus of interest. CE with conductometric detection was used to determine the total dissolved inorganic carbon in autonomous measurement devices [150,151]. CE can also be coupled with colorimetry [152] and amperometry [153] for the detection of a variety of inorganic analytes.

In addition, and complementary to CE, a wide array of electrochemical transduction methods for inorganic compounds exists [154]. The advantages of microfabrication coupled with drastically reduced cost of mass production and implementation offer these sensors the potential to access spatial and temporal niches that other sensing modes are not able to offer.

#### 3.4.4. Energy Redox Couples

Electrodes can be used to induce redox reactions to sense ion concentration. Ionic species have an electrical potential under which they can undergo part of a redox reaction. By applying this potential across electrodes in solution, a transfer of electrons between the electroactive species and the electrode occurs, resulting in a flow of the electrical current proportional to the concentration of the ionic species at the electrode surface [76]. Techniques that apply a varying potential are termed voltammetry, while those that apply a constant potential are termed amperometry.

In relation to astrobiology, these methods are particularly useful for the detection of species important to chemolithotrophic redox couples such as Mn (II) [155], with nanomolar lower limits of detection. Table 3 shows examples of amperometric or voltametric techniques applied to electrode designs to sense chemicals used in some chemolithotrophic and chemoorganotrophic processes. We note that many half-reactions useful to life in the form of ionic species have already been included in the discussion of sensing modalities, as they are either CHNOPS elements or are important in defining bulk ionic conditions in fluids.

Of these, oxygen is a common electron acceptor in chemolithotrophic redox reactions, although in many extraterrestrial environments, it will not be relevant, as they are generally anoxic. Perhaps the most widely used dissolved oxygen sensor to date is the Clark sensor. Named after one of its pioneers [167], the sensor was originally developed for monitoring oxygen levels in blood. Improvements in material and design have allowed this form of sensor to be used in environments with greater levels of interfering species [168]. This has in turn paved the way for wide-scale adoption of use in environmental monitoring; notably, in the case of habitability studies, their deployment with a suite of other sensors for studying the microenvironment of a shallow water hydrothermal vent [169]. Advances in microfabrication techniques have allowed for the reliable and inexpensive manufacture of planar microelectrodes [170,171]. This makes integration with other sensors possible [171], as well as with microfluidic sample delivery and containment systems [172,173].

**Figure 1:**
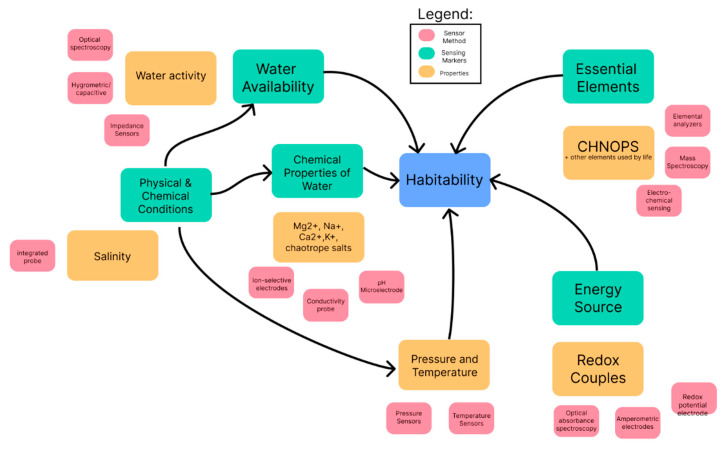
Key requirements for a Total Habitability Instrument (THI).

Figure 1 illustrates the essential indicators of the Total Habitability Instrument, highlighting how each required property is linked to its sensing method and marker.

## 4. Deployment—Integration with Soft and Bioinspired Systems

This review has highlighted some of the potential challenges of deploying the necessary sensors proposed for a THI into a space environment. Following this, consideration should be given to the challenges associated with the design and requirements for the locomotion platforms, including power requirements, weight implications, and cost comparisons. Considering the extreme environment of space, extreme temperature ranges, high-energy radiation, and varying and unpredictable terrain, these conditions present significant challenges to the operation and motion of space systems. Often, when we want habitability measurements, we want to gain them in situ in places that are potentially extreme and dynamic habitats. These can be difficult to access locations, such as the inside of rocks, underneath substrates, and within the liquid fractures of ice packs, etc. Soft robotics lends itself to addressing the challenges associated with the task of deploying a total habitability sensor within local physical conditions that require flexibility, dexterity, and the ability to deploy instruments in confined spaces. By deploying multiple individual soft robotic systems across a large area of interest, simultaneous sampling over large spatial areas can be achieved. Soft robotics, a field of research that is enormously multidisciplinary and uses alternative materials, such as soft materials, fluids, and biological components, can operate with centralised control architectures [174,175]. It is a field that is adopting new ways of designing and integrating smart machines using soft materials, providing potential for more cost-effective, robust systems. It is a field that is currently at the forefront of space exploration research.

Soft robotics often takes inspiration from natural biological systems, such as invertebrate organisms for construction and control. The animal kingdom has evolved to enable each species to adapt and operate in a variety of different dynamic and unstructured environments [176]. Hard robots are regularly ‘non-collaborative’ and can require predictable situations and accuracy. However, due to the design and control of soft robotics, they are inherently compliant and uniquely suited to extreme environments [177,178]. Compliance has advantages in many situations, including the requirement for gentle yet resilient and effective grippers for delicate objects or for use in dynamic and unstructured or extreme environments to perform various complex tasks. For example, when controlled by microfluidics, soft robotics become resilient to radiation [179].

Nature and its animals continuously demonstrate effective interactions with their surroundings through features such as configurability, compliance, and softness [176,180]. This is particularly true with animals that live in or around sand. An excellent example of this is the sandfish [181]. However, a robot moving through air or water will move fundamentally differently, for instance, in soil or a granular medium such as sand. The physics of movement in terradynamic locomotion are not the same as water or air, as the resistive drag forces are significantly greater, and the object in motion can be diverted from the course by the granular lift force [182,183,184]. A soft robot is able to conform to its surroundings due to its flexibility; however, the challenge is to design flexible actuation that is capable of high forces, which replicate the muscles in an animal’s body [185,186]. To be able to apply intentional forces to the individual task, the robot requires stiffness [187]. The solution to this challenge could lie in nature and how soft or relatively delicate animals provide this required stiffness to move through similar terrains, offering novel locomotion approaches [188,189]. Soft robotic systems have also drawn inspiration from creatures in nature that burrow [190,191], such as the razor clam [192], to use in the extreme environment of the offshore industry. Mack et al. have drawn inspiration from the physiology of a spider to develop a lightweight robotic system with significantly reduced energy requirements to address key challenges in extraterrestrial exploration associated with efficiency, controllability, and actuation efficiency [193].

Soft robotics has made significant scientific research progress in recent years. Research has investigated solutions to engineering challenges through the investigation of mimicking highly versatile locomotion of invertebrate organisms. However, there is significant research currently being conducted to understand embedding multiple sensing technologies into these soft materials [194,195,196,197,198,199]. Research involves expertise in advanced materials, flexible electronics, and fabrication methods.

**Figure 2:**
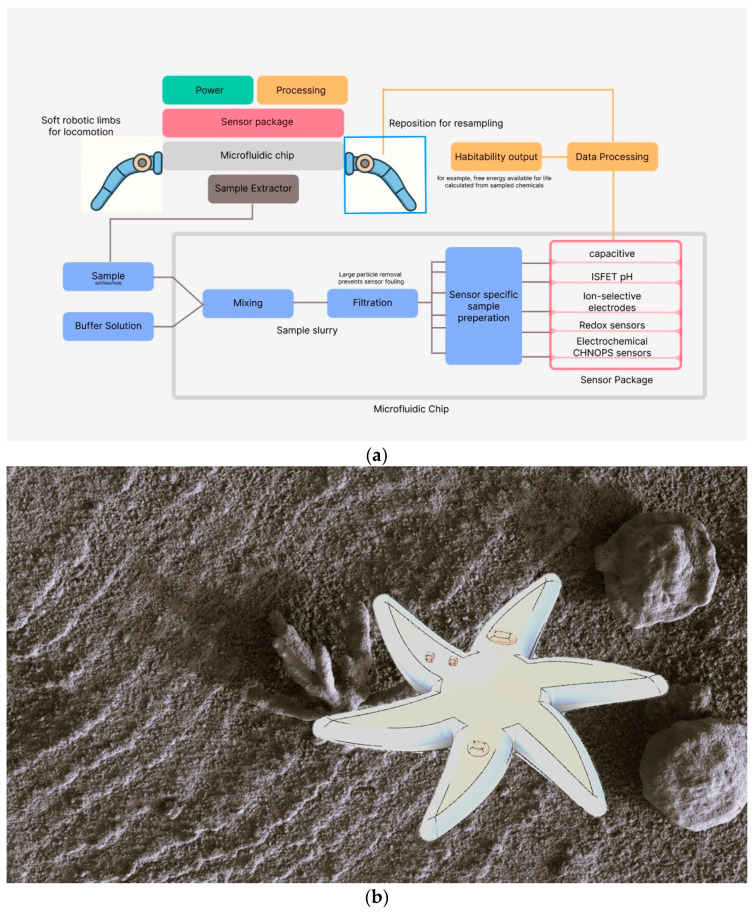
(**a**) Integration of THI robot with microfluidic devices. (**b**) Artistic rendition drawing inspiration from burrowing echinoderms of a soft robotic Total Habitability Instrument (THI) measurement system for planetary exploration. Capable of measuring conditions for habitability such as ions, water, and CHNOPS.

Advances in soft robotic locomotion and sensing integration, coupled with recent developments in soft additive manufacturing technologies [174,193,200], and the development and deployment of soft robotic platforms with integrated microfluidics for sensing provide promising potential, as shown in Figure 2.

## 5. A Total Habitability Instrument—Summary, Conclusions, and Conjecture

Based on the review of current sensing technologies and progress in the study of habitability, we make the case for research towards the development of a Total Habitability Instrument (THI). Established instruments such as MSs remain expensive and difficult to miniaturise, thus limiting their range of deployment. Smaller instruments that expand this range such as LIBS and Raman spectrometry are already used to complement MS capabilities with remote sensing and access to a different suite of analytes. Taking this trend further, we propose that a miniaturised total habitability system would be able to provide both novel and auxiliary data for future exploration.

Drawing inspiration from the design of *µ*TAS to include integrated microfluidics and electrochemical sensing for “backbone” habitability parameters, coupled with a distinct component for water activity measurements, microfluidics (lab-on-a-chip) is attracting attention for its potential in environmental research, as it allows for more precise manipulation of samples from the microscale to the nanoscale [201,202,203]. Some devices are integrated with Surface-Enhanced Raman Spectroscopy (SERS) [Emonds-Al]. The key objectives are to identify and analyse potential redox couples and to understand water activity, temperature, ionic strength, and the availability of important ionic species. It has been stated that the global miniature device market will hold a valuation of over 12–15 billion USD by 2030 [204]. Lab-on-a-chip technology has made considerable advances in the space sector [205], especially for the detection of analytes in extreme environments for space exploration [206,207]. Sniadek et al. propose that microfluidic systems, including lab-on-chip platforms, can be considered highly efficient for conducting any experiments, in space environments, that involve living organisms [208]. Additionally, with advancements in 3D printing, manufacturing of regulators, low-cost actuators, connectors, cost-effective construction materials, and adapters provide a platform to enable more compact system architectures. Ligtrink, TU Delft, developed a Life Marker chip (LMCool) to detect traces of life, specifically molecules in liquids, on Saturn’s moon Enceladus [209].

Ideally, such an instrument would be a single integrated, low-power, palmtop instrument, with the potential for low cost, to be deployed alongside larger systems or implemented alone in separate missions. The device would be autonomous, taking measurements and relaying data with minimal or no input from humans on the ground. It could be coupled with more significant sample extraction techniques, for example, similar to those proposed in the MOA instrument, to enable extraction from solid samples [41].

Such a device has the potential to be deployed in large numbers and integrated into simple soft robots. This integration could provide in-depth habitability over a geographical spread larger than a rover mission. The instruments could also occupy locations where continuous monitoring would enable an in-depth picture of temporal habitability. Multi-ion sensing chips consisting of microfabricated electrochemical sensors would allow for a certain “plug and play” capability, where one chip would be sensing for the major essential ions, while detection for the most likely redox couples could be added or subtracted depending on the likelihood of their occurrence.

As there are multiple parameters of interest, such a habitability instrument will include sensors with a high degree of integration capability. In this regard, thin-film microfabrication techniques would allow for a great number of these sensing modalities to be combined on a single, small transducing chip. Examples of this type of integration can be seen in, among other fields, sensing for environmental monitoring [66]. Sensing for conductivity and redox couples using micro- and nanoelectrodes, ion-selective electrodes, along with resistance devices for temperature sensing, could all be integrated on a single sensing chip. This would bring with it the key advantages of small size and weight, as well as the capability for multiple redundancies at little extra cost.

These could be integrated with sample delivery options, making use of microfluidics to sample small volumes of liquid from the environment of interest [210]. Due to the nature of these sensing modalities, they would require only small volumes of liquid to perform analysis, and as non-destructive analysis methods, they would not produce any waste material.

Some of the electrochemical sensors exhibit a narrower dynamic range, especially towards samples with higher concentrations of ions. This could be addressed by including some capability for sample processing, for example, dilution with deionised water. As much of the work to date has been focused on improving the lower limit of detection for these sensors, the capabilities of sensors to detect trace elements could compensate for the dilution.

This paper has identified the essential and comprehensive properties of a Total Habitability Instrument (THI) and highlighted promising approaches for its deployment. Future efforts should now focus on establishing key research and development priorities, particularly the miniaturisation of sensing technologies, the integration of lab-on-chip systems into soft architectures, and the definition of parameters tailored to specific mission requirements (for example, NASA’s ISRU), including environment (pressures, temperatures, and sample properties) and terrains [211]. Whilst developing bioinspired systems for operation in extreme environments, it is vitally important to align with required standards during its development. Cornejo et al. established a framework, Biomimetic Engineering and Aerospace Mechatronics Design (BEAM-DX), to formalise bioinspired design tools that are used in the advancement of autonomous planetary robotic systems deployed in deep space exploration scenarios. This framework integrates bioinspired behavioural, functional, and morphological properties with engineering protocols (ISO, VDI, and NASA’s TRL) [212].

The delivery of these sensors would depend on the environment in which they were placed. Interactions at the interfaces between water and rock release key elements into the wider liquid environment. This makes these areas a target for habitability studies, both for internal ocean worlds and for our own oceans [213]. Deep-sea exploration technology could be utilised in the case of internal ocean worlds such as Enceladus or Titan [214].

Many of these sensors will be affected by temperature and pressure. This could be compensated for by pre-calibration and inclusion of pressure sensors alongside the habitability sensor (as temperature would already be a parameter of interest). Making measurements in labs to characterise sensor responses at different temperatures and pressures will help to characterise their response once deployed. Many of the ion sensors also suffer from other sources of interference, such as interfering ions or pH, for many ISEs. The effects of these could in turn be minimised or factored out by the inclusion of several other ISEs to determine the presence and concentration of the interfering ions. This would greatly increase the reliability of a Total Habitability Instrument.

The proposed system is novel in that it constitutes a soft robotic platform with a comprehensive suite of integrated sensing components. Beyond existing multi-sensor systems used in past missions such as Phoenix, Curiosity, and ExoMars, its innovation lies in the integration of sensors and electronics within the THI. The design enables the deployment of multiple units over large areas, facilitating matrix sampling due to reductions in both mass and overall cost.

In this paper, key species to be measured and potential sensing modalities for a THI have been identified. From the diversity of measurements required within extreme environments, and from the advances made in soft robotic manufacturing, locomotion, and embedded systems, the integration of a THI remotely deployed by a soft robotic system would enable adaptable and diverse sensing opportunities.

## Figures and Tables

**Table 1:** Bioessential ions and examples of sensing options.

Ion	Examples of Biological Functions	Examples of Sensor
Na^+^	Osmotic balance, maintaining electrolytic balances, stability of molecules and structures	ISE with silver nanoparticles as solid contact [72]; ISFET array [73]
K^+^	Osmotic balance, maintaining electrolytic balances, stability of molecules and structures	ISE with silver nanoparticles as solid contact [72]; ISE with microporous carbon as solid contact [74]; ISFET array [73]
Mg^2+^	Stability of molecules and structures; essential cation pair for anions, e.g., phosphates	Solid contact pencil graphite electrodes with polypyrrole conducting polymer as solid contact [75]; carbon paste solid contact electrodes [76]
Ca^2+^	Outer cell membranes and coats in prokaryotes; wider array of functions in advanced eukaryotes and multicellular organisms	ISE array for cubesat experiment [77]; ISFET array [73]
Cl^−^	Osmotic balance, maintaining electrolytic balances, stability of molecules and structures	Amperometric electrochemical sensors [78]; ISE with silver nanoparticles as solid contact [72]

**Table 2:** Comparison of sensing methods.

Sensing Technology	Sensing Target	Technology Development	Minimum Size	Integration Capability
Glass electrodes	pH, some cations	Established	Order of ∼10 cm, plus readout instrument	Low
Liquid junction polymermembrane ISE	Major cations, e.g., K^+^, Mg^2+^, Ca^2+^	Established	Order of ∼10 cm, plus readout instrument	Low
Solid contact ISE	Major ions, e.g., K^+^, Mg^2+^, Ca^2+^	Some commercial availability, highly active research	Order of 10– 100 microns, miniaturised readout instrumentation	High
ISFET	Major cations, e.g., K^+^, Mg^2+^, Ca^2+^, pH	Commercial availability, highly active research	Order of <10 microns, miniaturised readout instrumentation	High

**Table 3:** Examples of electrochemical sensors for biologically relevant redox processes.

Chemical	Biological Redox Process	Examples of Electrochemical Sensor (Reference)
H_2_	Methanogenesis and H_2_ oxidation	Gold- and ceramic-based electrodes for measurement at high temperature and pressure [156]
CO_2_	Methanogenisis	Potentiometric measurements with ion-selective electrodes
O_2_	Several chemolithotrophic and chemoorganotrophic processes	Clark type sensors [157] and citations therein
Fe(III)	Fe reduction	Gold-modified carbon microelectrode ensemble [158] Thick-film-modified graphite electrode [159]
H_2_S	Oxidation of reduced S- species	Gold- and ceramic-based electrodes for measurement at high temperature and pressure [156] Carbon nanotube-modified glassy electrodes [160]
NO^2−^	Nitrite oxidation and anoxic ammonium oxidation	Cellulose-modified platinum electrodes [161]
NO^3−^	Nitrate reduction	Cellulose-modified platinum electrodes [161] Silver microelectrode with miniaturised sensing system [162]
Fe(II)	Fe oxidation	Au/Hg microelectrodes [163] Gold-modified carbon microelectrode ensemble [158]
Co	Trace metal oxidations and trace metal reductions	Bismuth microelectrode array [164]
As	Trace metal oxidations and trace metal reductions	Gold nanoelectrode ensembles [165] Gold-modified carbon screen-printed electrodes [166]
Mn	Trace metal oxidations and trace metal reductions	Voltammetric microelectrodes [163] Vibrating gold microwires [155]

## Data Availability

No new data were created or analyzed in this study.

## Abbreviations

The following abbreviations are used in this manuscript:

Beam-D	Biomimetic Engineering and Aerospace Mechatronics Design)
CE	Capillary electrophoresis
CHEMCAM	Chemistry and camera complex
CHNOPS	Carbon, Hydrogen, Nitrogen, Oxygen, Phosphorous, Sulphur
CMOS	Complementary metal–oxide semiconductor
EOA	Enceladus organics analyser
ERH	Equilibrium relative humidity
FETS	field effect transistor
INMS	Ion and neutral mass spectrometer
ISFETS	Ion-selective membrane field effect transistors
ISE	Ion-selective electrodes
ISO	International Organization for Standardization
ISM	Ion-selective membrane
IrOx	Iridium Oxide
JUICE	Jupiter icy moons explorer
LAKI	Life as we know it
LIBS	Laser-induced breakdown
LIF	Laser-induced fluorescence
MEMS	Microelectromechanical system
MOMA	Mars organic molecule analyser
MOSFET	Metal–oxide–semiconductor FET
MS	Mass spectrometer
PAHs	Polycyclic aromatic hydrocarbons
SERS	Surface-enhanced Raman spectroscopy
SPAD	Single-Photon Avalanche Diode
THI	Total habitability instrument
TRL	Technology Readiness Level
VDI	Verein Deutscher Ingenieure
